# Cytoplasmic FLIP(S) and nuclear FLIP(L) mediate resistance of castrate-resistant prostate cancer to apoptosis induced by IAP antagonists

**DOI:** 10.1038/s41419-018-1125-5

**Published:** 2018-10-22

**Authors:** Christopher McCann, Nyree Crawford, Joanna Majkut, Caitriona Holohan, Chris W. D. Armstrong, Pamela J. Maxwell, Chee Wee Ong, Melissa J. LaBonte, Simon S. McDade, David J. Waugh, Daniel B. Longley

**Affiliations:** Centre for Cancer Research and Cell Biology, School of Medicine, Dentistry and Biomedical Science, Queen’s University Belfast, Northern Ireland, UK

## Abstract

Expression of tumor necrosis factor-α (TNFα) in the serum of prostate cancer patients is associated with poorer outcome and progression to castrate-resistant (CRPC) disease. TNFα promotes the activity of NFκB, which regulates a number of anti-apoptotic and proinflammatory genes, including those encoding the inhibitor of apoptosis proteins (IAPs); however, in the presence of IAP antagonists, TNFα can induce cell death. In the presence of recombinant or macrophage-derived TNFα, we found that IAP antagonists triggered degradation of cIAP1 and induced formation of Complex-IIb, consisting of caspase-8, FADD and RIPK1 in CRPC models; however, no, or modest levels of apoptosis were induced. This resistance was found to be mediated by both the long (L) and short (S) splice forms of the caspase-8 inhibitor, FLIP, another NFκB-regulated protein frequently overexpressed in CRPC. By decreasing FLIP expression at the post-transcriptional level in PC3 and DU145 cells (but not VCaP), the Class-I histone deacetylase (HDAC) inhibitor Entinostat promoted IAP antagonist-induced cell death in these models in a manner dependent on RIPK1, FADD and Caspase-8. Of note, Entinostat primarily targeted the nuclear rather than cytoplasmic pool of FLIP(L). While the cytoplasmic pool of FLIP(L) was highly stable, the nuclear pool was more labile and regulated by the Class-I HDAC target Ku70, which we have previously shown regulates FLIP stability. The efficacy of IAP antagonist (TL32711) and Entinostat combination and their effects on cIAP1 and FLIP respectively were confirmed in vivo, highlighting the therapeutic potential for targeting IAPs and FLIP in proinflammatory CRPC.

## Introduction

Inflammation contributes towards the initiation and progression of prostate cancer^1^, with levels of inflammatory cytokines, such as tumor necrosis factor-alpha (TNFα), correlating with poor outcome and progression to castrate-resistant disease (CRPC)^2,3^. TNFα derived from cells in the tumor microenvironment can activate proinflammatory and pro-survival pathways in tumor cells, such as those mediated by the NFκB transcription factor family. Binding of TNFα to TNF-receptor 1 (TNFR1) results in formation of Complex-I, which contains receptor-interacting protein kinase-1 (RIPK1) and the cellular inhibitors of apoptosis proteins-1/2 (cIAP1/2). Within Complex-I, RIPK1 ubiquitination is mediated by cIAP1/2, subsequently leading to activation of NFκB^4^. Transcribed NFκB target genes, including those encoding anti-apoptotic proteins, such as cIAP1/2 and FLIP, and inflammatory cytokines, such as IL-8 and TNFα itself, act to further potentiate localized inflammation and cell survival^5^. In a previous study, we demonstrated that FLIP expression is elevated in CRPC and antagonizes response to androgen receptor-targeted therapy^6^.

Therapeutic IAP antagonists, such as TL32711 (Birinapant), have been developed based on the IAP-binding motif (Ala-Val-Pro-Ile) of the endogenous inhibitor of IAPs – SMAC (Second Mitochondrial-Derived Activator of Caspases) – and interact with the structurally conserved BIR (baculovirus IAP repeat) domains of IAPs^7^. IAP antagonist binding to the BIR domains of cIAP1 induce dimerization of its RING domains, stimulating E3-Ubiquitin ligase activity and subsequent auto-ubiquitination and proteasomal degradation of cIAPs^8^. cIAP1 depletion following IAP antagonist treatment leads to formation of a cytoplasmic cell death-regulating platform termed Complex-IIb, consisting of RIPK1, FADD and procaspase-8^9^. Procaspase-8 homodimerization at this complex results in its processing and activation, leading to downstream activation of caspases-3/7. Hetero-dimerization of procaspase-8 with either the long (FLIP(L)) or short (FLIP(S)) splice forms of FLIP in Complex-IIb inhibits procaspase-8 processing and therefore induction of apoptosis^10^. IAP antagonists can also disrupt the interaction between XIAP and caspases-3, -7 and -9^11,12^, thus relieving XIAP-mediated repression of these caspases and promoting the execution phase of apoptosis^13^.

TL32711 is a bivalent IAP antagonist which initially appeared promising in Phase1/2 clinical trials, but was later revealed to offer minimal clinical benefit to patients as a single agent and may act best alongside chemotherapeutic agents^14,15^. This has paved the way for the development of more potent IAP antagonists with improved bioavailability. The monovalent IAP antagonist ASTX660 is a non-peptidomimetic agent generated by structure-based design with potent on-target activity and favourable tolerability profile compared to bivalent peptide mimetic IAP antagonists and is currently in clinical development (Phase 1/2)^16^. In this study, we tested the hypothesis that proinflammatory, TNFα-rich, CRPC^3^ would be highly sensitive to IAP antagonists, as these agents convert this proinflammatory, anti-apoptotic cytokine into a cell death-inducing ligand.

## Materials and methods

### Compounds

TL32711 and Entinostat were obtained from Selleck Chemicals (Newmarket, UK), ASTX660 was obtained from Astex Pharmaceuticals (Cambridge, UK), z-VAD-fmk and Necrostatin-1 were purchased from Sigma-Aldrich (Gillingham, UK), GSK’874 and Necrosulfonamide from Merck (Darmstadt, Germany), recombinant TNFα from Alomone Labs (Israel), TNFα-neutralising antibody from Cell Signaling Technologies (Danvers, MA, USA) and Leptomycin-B solution was purchased from Sigma-Aldrich.

### Cell lines

PC3, DU145, VCaP and THP-1 cells were obtained from American Type Culture Collection (ATCC, Manassas, VA, USA) PC3, DU145 and THP-1 cells were cultured in RPMI medium (Invitrogen, Paisley, UK) with 10% fetal bovine serum (Invitrogen), and VCaP cells were cultured in DMEM (ATCC, LGC Standards, Middlesex, UK) with 10% fetal bovine serum.

### Generation of overexpressing cell lines

PC3 cell lines overexpressing wild-type and mutant FLIP spliceforms were generated as previously described^17^.

### Generation of PC3 CRISPR caspase-8 cell lines

PC3 CRISPR caspase-8 cells were generated by retroviral infection with pLentiCRISPRV2 with guide RNA AAGTGAGCAGATCAGAATTG which was provided as a kind gift from Prof. Galit Lahav, as described previously^18^. A mixed colony of cells was established following selection in Puromycin.

### Macrophage polarisation and conditioned-media collection

THP1 cells were differentiated to M1 polarised macrophages by 6 h treatment with 320 nM Phorbol 12-myristate 13-acetate (Sigma-Aldrich), followed by 18 h with 100 ng/mL Lipopolysaccharide (Sigma-Aldrich) and 20 ng/mL Interferon-γ. Conditioned media was collected after 24 h and incubated for 1 h with 100 ng/mL TNFα neutralizing antibody (Cell Signaling Technologies), where appropriate.

### Western blotting and subcellular fractionations

Western blotting was carried out as previously described^19^. Nuclear/Cytoplasmic fractionations are described in detail in Supplementary methods 1. cIAP1- and cIAP2-specific antibodies were from Enzo (Exeter, UK). XIAP, RIPK1, Acetylated-Lysine, HDAC1- and caspase 3-specific antibodies were from Cell Signaling Technology (Danvers, MA, USA). Caspase 8 antibody was from Alexis Biochemicals (San Diego, CA, USA). FLIP antibody was from Adipogen (San Diego, CA, USA). PARP antibody was from eBioscience (San Diego, CA). FADD antibody was obtained from BD Transduction Laboratories (Franklin Lakes, NJ, USA). Ku70 and HSP90 antibodies were from Santa Cruz Biotechnology (Dallas, Texas, USA). Secondary horseradish peroxidase-conjugated antibodies (Amersham, Buckinghamshire, UK) were used for detection on a G-Box digital developer (Syngene. Cambridge, UK).

### Flow cytometry

Analysis of cell-surface TNFR-1 expression was carried out on a BD Facs Caliber flow cytometer using the Cell Quest Pro software (BD Biosciences, San Diego, CA, USA), and cells stained using Phycoerythrin-conjugated anti-TNFR1 antibody (R&D Systems, Minneapolis, MN, USA) compared to an isotype control antibody. Annexin-V/Propidium Iodide flow cytometry was carried out on a BD LSR-II flow cytometer (BD Biosciences, San Diego, CA, USA) using FITC-Tagged Annexin-V (BD-Biosciences) and Propidium Iodide (Sigma-Aldrich).

### High-content Microscopy

High-content microscopy was carried out on an Array Scan XTI high content microscope (Thermo Scientific) using FITC-Tagged Annexin-V (BD-Biosciences) Propidium Iodide (Sigma-Aldrich), and Hoescht Stain (Invitrogen).

### siRNA transfections

All siRNAs (SC, cIAP2, XIAP, FLIP (L, S&T), RIPK1, Ku70) were obtained from Dharmacon (Chicago, IL, USA), and transfections carried out using Lipofectamine RNA iMAX (Life Technologies), as previously described^20^. Sequences shown in Supplementary methods 2.

### Immunoprecipitation

For complex-IIb immunoprecipitation cells were lysed in CHAPS buffer (30 mM Tris pH 7.5, 150 mM NaCl, 1% CHAPS). Anti-p18-capsase-8 antibody (1 μg, Santa Cruz, CA) was conjugated to 30 μL Protein G Dynabeads (Invitrogen, Paisley, UK). For acetylated-lysine immunoprecipitation, cells were lysed in RIPA buffer and 1 μg of acetylated-lysine antibody (Cell Signaling Technologies) was conjugated to beads. 750 μg of protein lysate was immunoprecipitated for 6 h at 4°C. IgG isoytpe controls were purchased from Santa Cruz. Co-Immunoprecipitation experiments were analysed by Western Blotting.

### Caspase 3/7 activity assay

Caspase 3/7 activity was assayed using Caspase-Glo® 3/7 assay according to manufacturer’s instructions (Promega, Madison, WI).

### Cell viability assay

Cell viability was assessed by Cell Titre-Glo® assay according to manufacturer’s instructions (Promega, Madison, WI, USA).

### In vivo xenograft study

All animal experiments were conducted in compliance with institutional guidelines and authority regulations. For in vivo anti-tumor efficacy study 1 × 10^6^ PC3 cells in 1:1 Matrigel:PBS were subcutaneously injected into male Balb/c SCID mice, one tumor per mouse. After tumors were established (volume ≥ 100 mm^3^), mice were treated with Vehicle (Veh) (1% DMSO in Peanut Oil (IP) and 30% Cyclodextrin (PO)), intraperitoneally with 20 mg/kg TL32711, orally with 15 mg/kg Entinostat (ENT), or with the TL32711 and Entinostat combination. Tumor volumes and animal weights were measured regularly until study endpoint (volume ≥ 500 mm^3^). Tumor tissues and blood were harvested for further analysis.

### Immunohistochemistry and Immunofluorescence

Immunohistochemistry staining on FFPE xenograft tissue for FLIP and cleaved-caspase-3 was carried out as previously described^21^. FLIP antibody was obtained from Abcam (Cambridge, England, UK) and used at a dilution of 1:1000, cleaved caspase-3 antibody was obtained from Cell-Signaling Technologies and used at a dilution of 1:200. Microscopy slides were digitally scanned using an Aperio CS2 slide scanner (Leica Biosystems, Milton Keynes, UK). Immunofluorescence was carried out using a Zeiss Apotome microscope. F4/80 antibody was obtained from Biorad (CA, USA) and used at a dilution of 1:200, Cytokeratin-5 antibody was used at a dilution of 1:200, and fluorescent labelled secondary antibodies from Invitrogen (CA, USA) used at 1:1000. Mounting media containing DAPI (Invitrogen) was used to fix coverslips and stain nuclei simultaneously.

### TNFα ELISA

Human TNFα was quantified by human TNFα ELISA (Abcam, Cambridge, UK), and murine TNFα (Abcam) from mouse serum as-per manufacturer’s instructions.

### Statistical Analysis

Experimental results were compared using a two-tailed Students t-test, or Two-Way ANOVA with Bonferroni Post-test, where appropriate. Experiments were carried out in triplicate, values represent mean ± SEM. *p ≤ 0.05 **p ≤ 0.01 and ***p ≤ 0.001.

## Results

### IAP antagonists have rapid and potent on target activity in prostate cancer cell lines

**Fig. 1:**
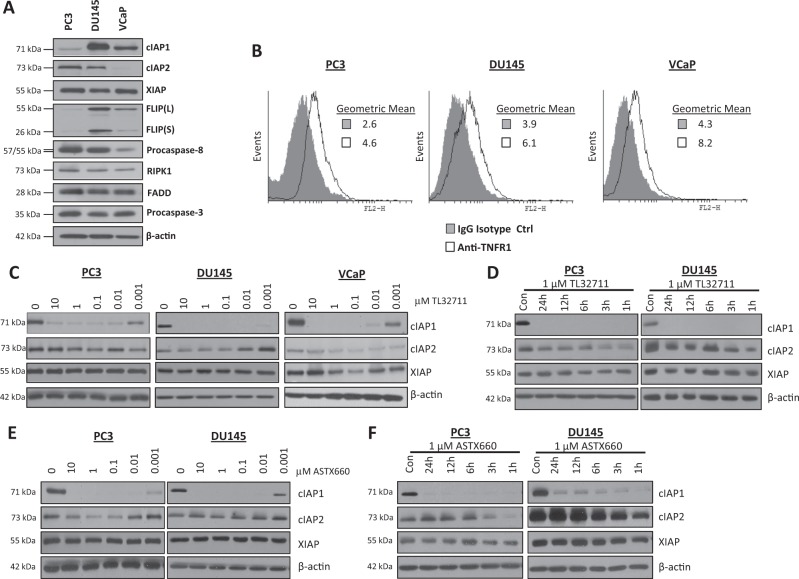
IAP antagonists have rapid and potent on target activity in prostate cancer cell lines. **a** Western blot analysis of basal expression of cIAP1, cIAP2, XIAP, FLIP(L), FLIP(S), Procaspase-8, RIPK1, FADD, Procaspase-3 and β-actin in PC3, DU145 and VCaP cell lines. **b** Flow cytometric analysis of basal cell surface expression of TNFR1 in PC3, DU145 and VCaP cell lines compared to an IgG isotype control. **c** Western blot of cIAP1, cIAP2 and XIAP expression following treatment with 0, 10, 1, 0.1, 0.1, 0.01 and 0.001 μM TL32711 in PC3, DU145 and VCaP cell lines for 24 h. **d** Western Blot of cIAP1, cIAP2, and XIAP expression following 1, 3, 6, 12 and 24 h treatment with a clinically-achievable dose of 1 μM TL32711 in PC3 and DU145 cells. **e** Western blot analysis of cIAP1, cIAP2 and XIAP expression following treatment with 0, 10, 1, 0.1, 0.1, 0.01 and 0.001 μM ASTX660 in PC3, and DU145 cell lines for 24 h. **f** Western Blot of cIAP1, cIAP2, and XIAP expression following 1, 3, 6, 12 and 24 h treatment with 1 µM ASTX660 in PC3 and DU145 cell lines

PC3, DU145 and VCaP cell lines were used throughout this study as they represent CRPC^22^. VCaP cells are a truly castrate-resistant model as, although they express androgen receptor, they do not respond to androgen-deprivation therapy in vitro or in vivo^23^. Core components of the TNFR1 apoptosis signaling pathway were profiled at the protein level by Western blot (Fig. 1a) and flow cytometry (TNFR1; Fig. 1b). cIAP1 expression was high in DU145 and VCaP, whereas cIAP2 expression was highest in PC3 cells and absent in the VCaP model. XIAP was expressed at a similar level in the three models. Expression of the Complex-IIb adaptors RIPK1 and FADD were similar across the models as was expression of procaspase-3; however, procaspase-8 expression was significantly lower in the VCaP model. Notably, expression of both FLIP splice forms correlated with expression of cIAP1 in the 3 cell lines. Similar levels of cell-surface TNFR1 were detected in each cell line (Fig. 1b).

The bivalent IAP antagonist TL32711^24^ induced degradation of cIAP1 in PC3, DU145 and VCaP cells (Fig. 1c); a widely accepted pharmacodynamic (PD) biomarker of on-target activity for this class of agent^25^ and confirms potent on-target activity of TL32711 at nanomolar concentrations. Of note, no consistent effect on XIAP expression was observed. In timecourse studies, rapid depletion of cIAP1 was observed in response to TL32711 (Fig. 1d); cIAP2 downregulation was also detected at early timepoints, but recovered to near control levels after 3 h, consistent with reports of IAP antagonist-induced activation of the non-canonical NFκB pathway following cIAP downregulation and the requirement for cIAP1 to mediate cIAP2 ubiquitination and degradation^26–28^. In contrast, XIAP levels remained relatively unaffected by TL32711. Similar results were obtained with the monovalent IAP antagonist ASTX660 (Fig. 1e–f).

### Prostate cancer cell lines are resistant to TL32711 + TNFα combination

**Fig. 2:**
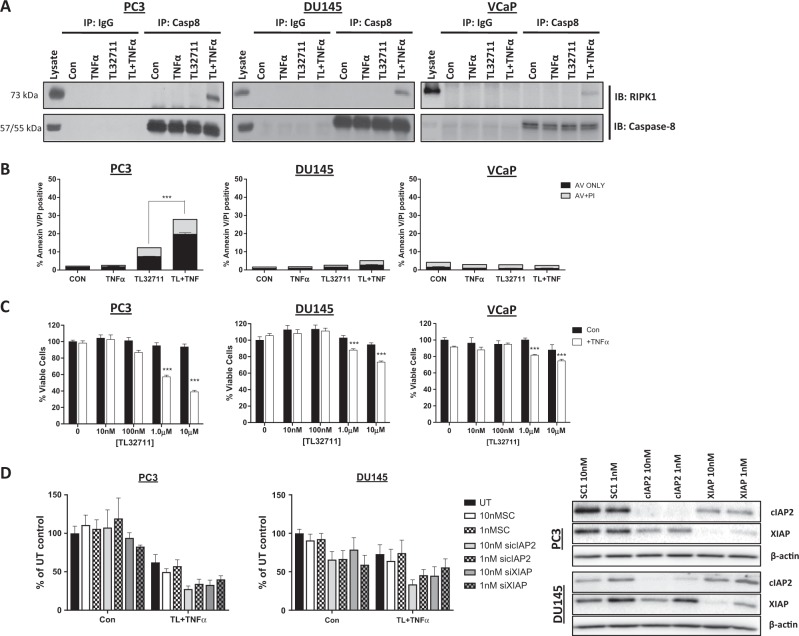
Prostate cancer cell lines are resistant to TL32711 + TNFα combination. **a** Western blot of RIPK1 and caspase-8 following caspase-8 immunoprecipitation in PC3, DU145 and VCaP cells treated for 3 h with 1 µM TL32711 in the presence and absence of 10 ng/mL TNFα; cells were pretreated with 20 µM z-VAD-fmk. **b** Annexin-V/Propidium Iodide flow cytometry analysis of PC3, DU145 and VCaP cells treated for 24 h 1 µM TL32711(TL) + /−10 ng/mL TNFα. **c** Cell viability assay in PC3, DU145 and VCaP cell lines treated for 72 h with 10 nM, 100 nM, 1 μM or 10 μM + /−10 ng/mL TNFα. **d** Cell Viability assay in PC3 and DU145 cells untransfected (UT) or pre-treated for 24 h with 10 or 1 nM Scrambled Control (SC1),cIAP-2, or XIAP siRNA followed by 48 h 1 μM TL32711 + 10 ng/mL TNFα. *p ≤ 0.05, **p ≤ 0.01 and ***p ≤ 0.001

Following treatment with a clinically-achievable dose 1μM TL32711 for 3 h in the presence of 10 ng/mL recombinant TNFα (to model a proinflammatory microenvironment), formation of TNFR1 Complex-IIb was observed in all cell lines as indicated by interaction between caspase-8 and RIPK1 (Fig. 2a). Only in the PC3 model did formation of Complex-IIb correlate with downstream activation of significant levels of apoptosis (Fig. 2b). TL32711 alone induced a small but consistent increase in cell death in PC3 cells, although neither this line nor DU145 secreted detectable levels of TNFα basally or following IAP antagonist treatment as determined by ELISA (Supplementary Figure 1). Recombinant TNFα alone had no effect on cell death in any of the CRPC models. These results were mirrored in cell viability assays, with DU145 and VCaP cells exhibiting profound resistance (over 80% cell viability relative to control in response to 1μM TL32711 plus TNFα; Fig. 2c). Even in the more sensitive PC3 model, ~60% cell viability relative to control was observed in response to 1μM TL32711 in combination with TNFα, and almost no activity with TL32711 monotherapy was observed. Similar results were obtained with ASTX660 (Supplementary Figure 2A-B).

Taken together, these findings suggest that, despite efficient formation of Complex-IIb, CRPC is inherently resistant to IAP antagonist-induced apoptosis, even in the presence of TNFα. As cIAP1 is rapidly and potently (1 nM) downregulated by both IAP antagonists in these models, we assessed potential for resistance mediated by the other 2 main IAPs, cIAP2 and XIAP, using RNAi. Downregulation of either cIAP2 or XIAP (Fig. 2d) failed to significantly enhance sensitivity to TL32711/TNFα in PC3 and DU145 cell lines, suggesting that other factors are involved in mediating resistance to IAP antagonists in CRPC.

### Cytoplasmic FLIP(S) and nuclear FLIP(L) mediate resistance of CRPC to IAP antagonist-induced apoptosis

**Fig. 3:**
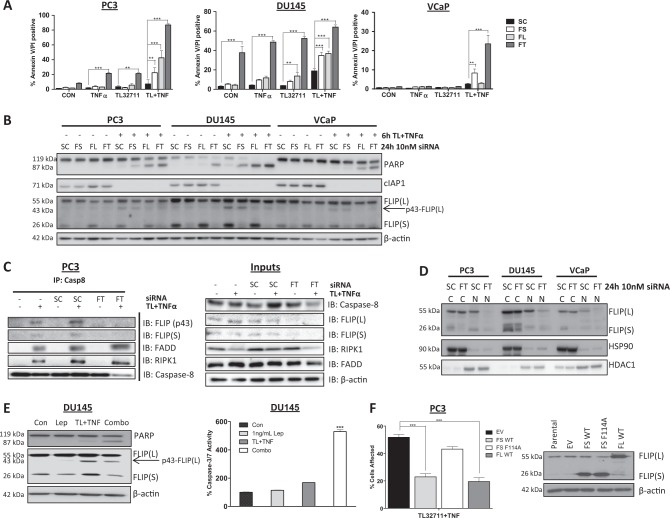
Cytoplasmic FLIP(S) and nuclear FLIP(L) mediate resistance of CRPC to apoptosis induced by IAP antagonists. **a** Annexin-V/PI high content microscopy analysis of PC3, DU145 and VCaP cells treated for 24 h with 10 nM scrambled control (SC) siRNA, FLIP(S) specific (FS) siRNA, FLIP(L)-specific (FL) siRNA or siRNA targeting both FLIP(L) and FLIP(S) spliceforms (FT) siRNA followed by 24 h treatment with 1 μM TL32711(TL) + /−10ng/mL TNFα. **b** Western blot of PARP, cIAP1 and FLIP expression in PC3, DU145 and VCaP cells treated for 24 h with 10 nM scrambled control (SC) siRNA, FLIP(S) specific (FS) siRNA, FLIP(L)-specific (FL) siRNA or siRNA targeting both FLIP(L) and FLIP(S) spliceforms (FT) siRNA followed by 6 h treatment with 1 μM TL32711 in combination with 10 ng/mL TNFα. **c**, Left – Western blot of FLIP, FADD, RIPK1 and caspase-8 following caspase-8 immunoprecipitation in PC3 cells treated for 24 h with 10 nM Scramble Control (SC), FLIP-Total (FT) or untransfected (−) followed by 3 h with 1 µM TL32711 and 10 ng/mL TNFα; cells were pretreated with 20 µM z-VAD-fmk. Total cell lysate is presented in the panel on the right. **d** Western blot of FLIP, HDAC1 and HSP90 in cytoplasmic (cC) and nuclear (nN) fractions following 24 h treatment with 10 nM scrambled control (SC) siRNA or siRNA targeting both FLIP(lL) and FLIP(sS) spliceforms (FT). **e** Western blot of PARP and FLIP expression (*Left*), and caspase-3/7 activity assay (*right*) in DU145 cells treated for 1 h with 1 ng/mL Leptomycin-B (Lep), 6 h TL32711(TL) + TNFα or pretreated for 1 h with Leptomycin-B(Lep) followed by 6 h TL32711(TL) + TNFα (Combo). **f** Cell viability assay of PC3 cells retrovirally infected to stably overexpress empty vector(EV), wild-type FLIP(sS)(FS WT), wild-type FLIP(L) (FL WT) and FADD-binding-deficient F114A mutant FLIP(sS) (FS F114) treated for 72 h with 1 μM TL32711 in combination with 10 ng/mL TNFα (Left). Right: Western blot analysis of parental PC3 and PC3 cells retrovirally infected to stably overexpress empty vector(EV), wild-type FLIP(sS)(FS WT), wild-type FLIP(L)(FL WT) and FADD-binding-deficient F114A mutant FLIP(sS)(FS F114A). *p ≤ 0.05, **p ≤ 0.01 and ***p ≤ 0.001

FLIP is an inhibitor of caspase-8 activation^29^, and the more resistant CRPC models (DU145 and VCaP) express significantly higher levels of both FLIP splice forms than the more sensitive PC3 model (Fig. 1a). Moreover, we have previously found that FLIP is frequently overexpressed in CRPC^6^. We therefore assessed FLIP’s role in mediating the observed resistance to IAP antagonists. In all three models, simultaneously downregulating both FLIP(L) and FLIP(S) by siRNA (FT) significantly enhanced cell death induction in response to TL32711/TNFα (Fig. 3a), and these effects were reflected in reductions in cell viability (Supplementary Figure 3). Similar effects were observed with the monovalent IAP antagonist, ASTX660 (Supplementary Figure 4). Notably, simultaneous downregulation of FLIP(L) and FLIP(S) with the dual splice form-targeted siRNA (FT) resulted in significant levels of apoptosis induction in the DU145 model (the model with highest FLIP levels) in the absence of co-treatment with either IAP antagonist or TNFα, indicating that this model is intrinsically FLIP-dependent. Also, in PC3 cells, simultaneous downregulation of FLIP(L) and FLIP(S) enhanced sensitivity to both TL32711 alone and TNFα alone, an effect also observed in DU145, but not VCaP cells. Specific silencing of either the long (FL siRNA) or the short (FS siRNA) splice form also enhanced cell death induction in response to TL32711/TNFα, although to a lesser extent than simultaneously targeting both splice forms, indicating that both FLIP splice forms mediate resistance (Fig. 3a).

Qualitative assessment of apoptotic cell death by PARP cleavage confirmed the results of the cell death and cell viability assays, with enhanced PARP cleavage observed after transfection with siRNA targeting both FLIP(L) and FLIP(S) splice forms-in combination with TL32711/TNFα in all three models (Fig. 3b). Transfection with the FLIP(L)-specific siRNA also enhanced TL32711/TNFα-induced PARP cleavage in all three cell lines, but to a lesser extent than the dual-targeted siRNA, while the FLIP(S)-specific siRNA enhanced TL32711/TNFα-induced PARP cleavage in PC3 and DU145 cells, albeit to a lesser extent than the FT and FL siRNAs. Despite the clear enhancement of sensitivity to TL32711/TNFα in FLIP siRNA transfected cells, there was a relative lack of downregulation of FLIP(L) in cells transfected with either the dual splice form targeting or selective FLIP(L) siRNA in all three models (Fig. 3b). In contrast, FLIP(S) was efficiently downregulated by both the FLIP(S)-specific and FT siRNAs. The p43-cleavage product of FLIP(L), which is generated when it heterodimerizes with procaspase-8 in Complex IIb^29^, was depleted in FT and FL siRNA transfected cells, suggesting that it is the pool of FLIP(L) which can be recruited to Complex-IIb that is effectively targeted by these siRNAs. Importantly, p43-FLIP was detected in complex-IIb (along with FADD, RIPK1 and FLIP(S); Fig. 3c), indicating that FLIP(L) is recruited to this complex and processed by caspase-8; importantly, p43-FLIP was not detected in complex-IIb in the FT siRNA transfected cells.

The FLIP long (FLIP(L)) splice form has been reported to possess nuclear import and export signals^30^; we therefore assessed whether either the cytoplasmic or nuclear pools of FLIP(L) were being targeted by the FL and FT siRNAs. We found that it was the nuclear pool of FLIP(L) that was downregulated (Fig. 3d), suggesting (somewhat surprisingly) that it is this pool of FLIP(L), which, along with the predominantly cytoplasmic FLIP(S), mediates resistance to TL32711/TNFα in CRPC cells. This conclusion was further supported by the finding that the nuclear export inhibitor Leptomycin-B reduced cleavage of FLIP(L) to p43-FLIP(L) (indicating that it abrogates recruitment of FLIP(L) to Complex-IIb) and enhanced TL32711/TNFα-induced PARP cleavage (Fig. 3e, Left) and caspase activation (Right).

Further support for the critical role of FLIP in modulating CRPC resistance to TL32711/TNFα was obtained using PC3 cell lines stably overexpressing wild-type FLIP(L), wild-type FLIP(S), or F114A mutant FLIP(S) (Fig. 3f, Right), which we have previously demonstrated binds inefficiently to FADD and therefore fails to prevent procaspase-8 homodimerization and activation at FADD-dependent complexes^31^. We found that overexpression of either wild-type FLIP protein, but not the F114A FLIP(S) mutant protected PC3 cells against TL32711/TNFα-induced cell death (Fig. 3f); this confirms the criticality of the FLIP/FADD interaction within Complex-IIb for FLIP’s ability to confer resistance.

### Entinostat downregulates FLIP and overcomes resistance to apoptosis induced by IAP antagonists

**Fig. 4:**
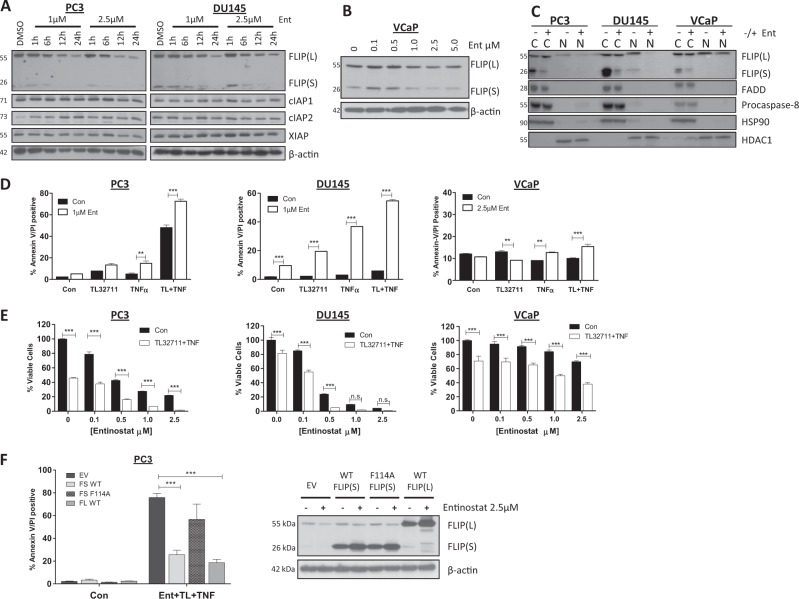
Entinostat downregulates FLIP and overcomes resistance to apoptosis induced by IAP antagonists. **a** Western Blot of FLIP, cIAP1, cIAP2, XIAP expression in PC3 and DU145 cells following 0 (DMSO), 1, 6, 12 or 24 h treatment with 1 or 2.5 μM Entinostat. **b** Western blot analysis of FLIP expression in VCaP cells following 24 h treatment with 0, 0.1, 0.5, 1.0, 2.5 or 5 μM Entinostat (Ent). **c** Western blot analysis of FLIP, FADD, Procaspase-8, HSP90 and HDAC1 in cytoplasmic (cC) and nuclear (nN) fractions following 24 h treatment with 2.5 μM Entinostat(Ent). **d** Annexin-V/PI flow cytometry analysis of PC3, DU145 and VCaP cells upon 24 h pre-treatment with 1 or 2.5 μM Entinostat (**EE**nt) followed by 24 h with 1 µM TL32711(TL) +/−10 ng/mL TNFα. **e** Cell viability assay following 24 h pretreatment with 0, 0.1, 0.5, 1.0 and 2.5 μM Entinostat followed by 48 h with TL32711 + TNFα. **f** Left: Annexin-V/PI flow cytometry analysis in PC3 cells retrovirally infected to stably overexpress empty vector(EV), wild-type FLIP(**sS**)(FS WT), wild-type FLIP(lL) (FL WT) and FADD-binding-deficient F114A mutant FLIP(**sS**) (FS F114) after 24 h 2.5 μM Entinostat(Ent) in combination with 1 µM TL32711(TL) and 10 ng/mL TNFα, Right: Western blot analysis of FLIP expression in in PC3 cells retrovirally infected to stably overexpress empty vector(EV), wild-type FLIP(S)(FS WT), wild-type FLIP(L) (FL WT) and FADD-binding-deficient F114A mutant FLIP(S) (FS F114) following 24 h 2.5 μM Entinostat *p ≤ 0.05, **p ≤ 0.01 and ***p ≤ 0.001

The second generation Class I-selective histone deacetylase (HDAC) inhibitor Entinostat has demonstrated clinical activity in hormone-resistant Breast Cancer^32^. In both the PC3 and DU145 CRPC models, we found that Entinostat downregulated both FLIP(S) and FLIP(L) expression, whereas expression of IAPs was unaffected (Fig. 4a). In the VCaP model, the effect of Entinostat on FLIP was more modest (Fig. 4b). Notably, Entinostat downregulated nuclear rather than cytoplasmic FLIP(L) in the PC3 and DU145 models, although it had almost no effect on nuclear FLIP(L) in the VCaP model (Fig. 4c). FLIP(S) was again found to be predominantly expressed in the cytoplasm and was downregulated by Entinostat. Other components of Complex-IIb (procaspase-8 and FADD) were expressed in the cytoplasm and were unaffected by Entinostat treatment. In addition, FLIP downregulation was mediated at the post-transcriptional level, as FLIP mRNA expression was not downregulated in response to Entinostat; in fact, both FLIP(L) and FLIP(S) mRNAs were upregulated in the DU145 model (Supplementary Figure 5).

The ability of Entinostat to enhance TL32711/TNFα-induced cell death correlated with its ability to downregulate nuclear FLIP(L) and cytoplasmic FLIP(S), as PC3 and DU145 models were sensitized to TL32711/TNFα, whereas VCaP cells remained relatively resistant, as assessed by cell death (Fig. 4d) and cell viability (Fig. 4e) assays. Similar results were again obtained with ASTX660 (Supplementary Figure 6). Furthermore, FLIP downregulation was confirmed to be critical for Entinostat to overcome resistance to IAP antagonist therapy: in PC3 cells overexpressing FLIP (either long or short splice forms), Entinostat was unable to downregulate the exogenous FLIP proteins (Fig. 4f, Right), consistent with our previous observations^33^. Subsequently PC3 cells overexpressing wild-type FLIP(S) or FLIP(L) were resistant to the TL32711/TNFα and Entinostat combination (Fig. 4f, Left). In contrast, PC3 cells overexpressing the FADD-binding mutant F114A FLIP(S) had comparable sensitivity to the EV control cells, confirming the essentiality for FLIP binding to FADD in Complex-IIb to block cell death induction in the context of Entinostat co-treatment.

### Entinostat enhances apoptosis in response to IAP antagonists via Ku70, RIPK1 and caspase-8

**Fig. 5:**
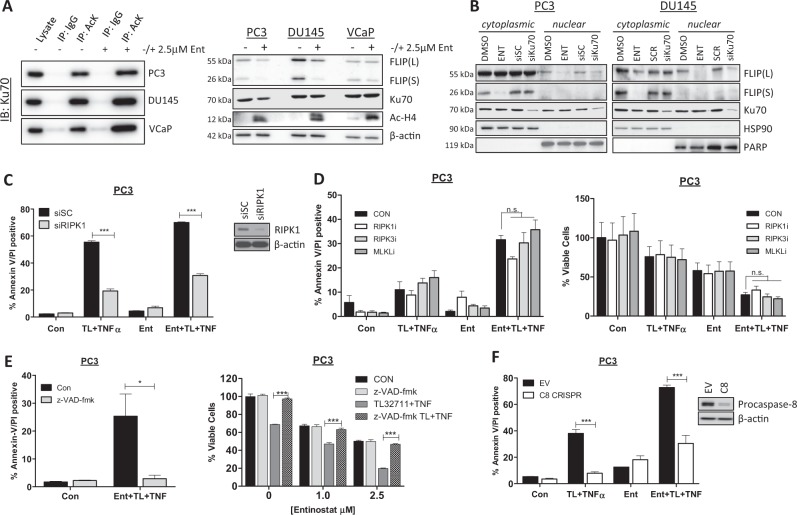
Entinostat enhances apoptosis in response to IAP antagonists via Ku70, RIPK1 and caspase-8. **a** Western blot of Ku70 in PC3, DU145 and VCaP cells following immunoprecipitation with anti-acetylated Lysine (AcK) or IgG isotype control antibody after 24 h treatment with 2.5 µM Entinostat(Ent) (Left). Right: Western blot of FLIP, Ku70, Hyperacetylated Histone4 (Ac-H4) in PC3, DU145 and VCaP cells treated for 24 h with 2.5 µM Entinostat (Ent). **b** Western blot of FLIP, Ku70 and HSP90 and PARP in nuclear and cytoplasmic fractions from PC3 and DU145 cells treated for 24 h with 2.5 μM Entinostat(ENT) or transfected with 20 nM scrambled control(SC) or Ku70 siRNA. **c** Annexin-V/PI flow cytometry analysis of PC3 cells transfected for 24 h with 20 nM scrambled control (SC) or RIPK1 siRNA followed by 24 h of 2.5 µM Entinostat(Ent) and a further 24 h with 1 µM TL32711 and 10 ng/mL TNFα combination. Western blot analysis of RIPK1 and β-actin in PC3 cells treated with 10 nM SC or RIPK1 siRNA. **d** Annexin-V/PI flow cytometry *(Left)*and cell viability assay (Right) in PC3 cells pre-treated for 1 h with 20 µM Necrostatin-1 (RIPK1 kinase inhibitor), GSK’840 (RIPK3 kinase inhibitor Necrosulfonamide (MLKL inhibitor) followed by pre-treatment for 24 h with 2.5 µM Entinostat(Ent) and a further 24 h with 1 µM TL32711 and 10 ng/mL TNFα combination. **e** Annexin-V/PI flow cytometry (Left) and cell viability assay (Right) in PC3 cells pre-treated for 1 h with 20 µM z-VAD-fmk followed by pre-treatment for 24 h with 2.5 µM Entinostat(Ent) and a further 24 h with 1 µM TL32711 and 10 ng/mL TNFα combination. **f** Annexin-V/PI flow cytometry analysis of PC3 empty vector(EV) and procaspase-8(C8) CRISPR cells following pre-treatment for 24 h with 2.5 µM Entinostat(Ent) and a further 24 h with 1 µM TL32711 and 10 ng/mL TNFα combination. *p ≤ 0.05, **p ≤ 0.01 and ***p ≤ 0.001

Given the multiple potential modes of action of HDAC inhibitors^34,35^, we further explored the mechanism by which Entinostat enhanced sensitivity to TL32711/TNFα. HDAC inhibition in colorectal cancer cells leads to the acetylation of the DNA damage repair protein Ku70, leading to the disruption of a stabilizing interaction between FLIP and Ku70, resulting in ubiquitin-mediated FLIP degradation^33^. To assess the role of Ku70 in regulating FLIP expression in prostate cancer cells, we first confirmed that Entinostat enhanced acetylation of Ku70 (Fig. 5a, Left) whilst also depleting FLIP expression in PC3 and DU145 cells (Fig. 5a, Right); subsequently, we demonstrated that, similar to Entinostat, Ku70 downregulation using siRNA caused depletion of nuclear but not cytoplasmic FLIP(L), although, unlike Entinostat, it had no effect on cytoplasmic FLIP(S) (Fig. 5b). These results suggest that nuclear FLIP(L) is stabilized by Ku70 in prostate cancer cells and that Ku70 acetylation in response to Entinostat results in downregulation of this subcellular fraction of FLIP(L).

IAP antagonists have been shown to induce cell death through Complex-IIb via two RIPK1-dependent mechanisms: caspase-8-mediated apoptosis and RIPK3-mediated necroptosis^36^. As expected given the observed formation of RIPK1/procaspase-8 complex in response to TL32711/TNFα (Figs 2a and 3c), RIPK1 siRNA significantly protected CRPC cells against cell death induced by TL32711/TNFα, both with and without Entinostat pre-treatment (Fig. 5c). Small molecule inhibition of RIPK1 or RIPK3 kinase activity or MLKL oligomerisation (with Necrostatin-1, GSK’840 or Necrosulfonamide, respectively) failed to protect against TL32711/TNFα and Ent/TL32711/TNFα-induced cell death (Fig. 5d). The pan-caspase inhibitor z-VAD-fmk completely protected against apoptosis and reduction in viability induced by Entinostat in combination with TL32711/TNFα (Fig. 5e). PC3 cells deficient in caspase-8 (PC3-C8 CRISPR) were protected against TL32711/TNFα with and without Entinostat, confirming that the cell death induced is caspase-8-dependent apoptosis in both the absence and presence of the HDAC inhibitor (Fig. 5f). Collectively these results indicate that the scaffold function, but not the kinase activity of RIPK1 is essential for the apoptotic cell death induced by this combination in CRPC.

### Macrophage-derived TNFa and in vivo- efficacy of TL32711

**Fig. 6:**
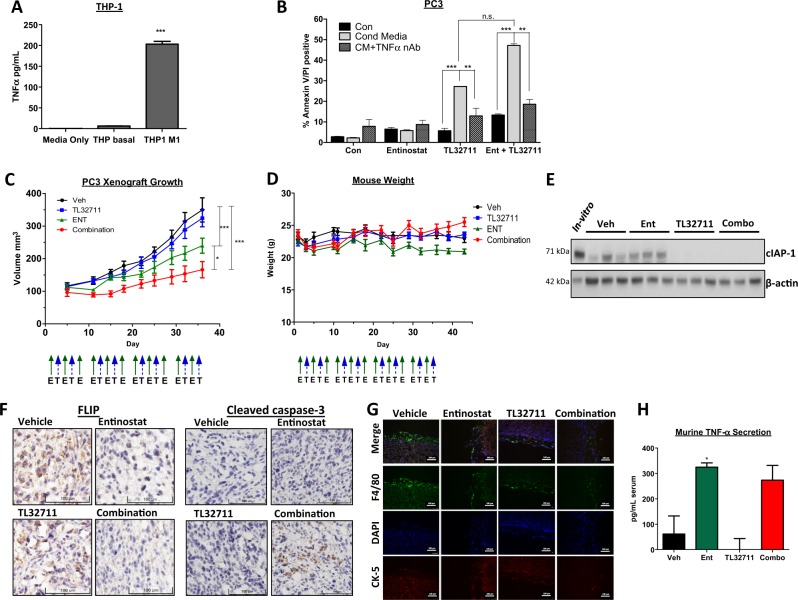
Macrophage-derived TNFα and in vivo efficacy of TL32711. **a** Human TNFα ELISA quantifying TNFα secretion from THP1 cells (THP basal) and M1-differentiated THP1 cells(THP M1) at 24 h. **b** Annexin-V/PI flow cytometric analysis of PC3 cells pretreated for 24 h with 2.5 μM Entinostat(Ent) followed by 24 h with 1 µM TL32711 cultured in M1-derived conditioned mediain the presence(CM + TNFα nAb) or absence(Cond Media) of 100 ng/mL TNFα neutralising antibody. **c** Tumor volume analysis of PC3 xenografts implanted into SCID-mice treated with Vehicle(Veh), 20 mg/kg TL32711, 15 mg/kg Entinostat(ENT) or TL32711 and Entinostat combination(schedule as indicated; E- Entinostat, T- TL32711). **d** Mouse weights for duration of xenograft study. *p ≤ 0.05, **p ≤ 0.01 and ***p ≤ 0.001. **e** Western blot analysis of cIAP1 expression in PC3 cells pre-implantation *(*in vitro*)* and three individual PC3 xenograft tumor lysate 24 h following final dose of Vehicle (Veh), Entinostat(Ent), TL32711 or Combination(Combo). **f** Immunohistochemical analysis of FLIP (Left), and cleaved caspase-3 (*Right*) expression in PC3 xenograft tumors from mice 24 h following final dose of Vehicle, Entinostat, TL32711 or Combination. Representative IHC images of 3 regions of 3 tumors collected per treatment group. **g** Immunoflurorescent microscopy of the murine macrophage marker F4/80, cytokeratin (CK)-5 epithelial cell marker, and DAPI-stained nuclei in PC3 xenograft tissue. Scale bar represents 100 μm. **h** Murine TNFα ELISA quantifying murine TNFα present in the serum of mice 24 h following final dose of Vehicle (Veh), Entinostat (Ent), TL32711 or Combination(Combo). Data represents pooled values in duplicate from 3 mice per treatment group. *p ≤ 0.05, **p ≤ 0.01 and ***p ≤ 0.001

To more closely mimic the proinflammatory tumor microenvironment (TME), we assessed the ability of macrophage-derived TNFα to induce apoptosis in the context of IAP inhibition. Differentiation of human THP-1 monocytic cells into M1-like macrophages significantly increased their secretion of TNFα (Fig. 6a). PC3 cells co-treated with M1 THP-1 conditioned media were sensitive to TL23711 alone, and this sensitivity was greatly increased in PC3 cells pre-treated with Entinostat (Fig. 6b). Importantly, use of a TNFα-neutralizing antibody demonstrated that the cell death induced in the presence of M1 THP-1 conditioned media was TNFα-dependent.

The strategy of using Entinostat to overcome resistance of CRPC to IAP antagonists was further explored in a PC3 xenograft study (Fig. 6c). In support of our in vitro findings, co-treatment with Entinostat and TL32711 retarded tumor growth more than treatment with either agent individually. Moreover, this combination was well tolerated (Fig. 6d). Depletion of cIAP1 in the TL32711 and combination treatment groups demonstrated TL32711 on-target activity (Fig. 6e). Moreover, immunohistochemistry analyses confirmed FLIP depletion in Entinostat-treated groups and increased levels of cleaved caspase-3 (a marker of apoptosis) in the combination-treated group (Fig. 6f). The presence of macrophages in close proximity to the engrafted tumor cells was confirmed by immunofluorescent microscopy (Fig. 6g); with positive staining for the murine-macrophage marker F4/80^37^ evident in all treatment groups. Such macrophages may act as the source of murine-derived TNFα detected in the serum of these mice, as quantified by ELISA in Fig. 6h. Notably, Entinostat, but not TL32711, significantly enhanced TME (murine)-derived TNFα, suggesting a further mechanism by which Entinostat can enhance sensitivity to IAP antagonists (Fig. 6h).

## Discussion

With chronic inflammation often being cited as a key driver of prostate tumorigenesis and disease progression^1^, high levels of circulating TNFα observed in CRPC patients^3^, and high intra-tumoral expression of TNFα and TNFR1 cited as being associated with poor clinical outcomes^38^, the potential for exploiting the pro-death activity of TNFα was investigated. We hypothesized that inhibiting IAPs would cause TNFα to induce cell death, thus providing a therapeutic rationale for using IAP antagonists in proinflammatory CRPC.

Both bivalent (TL32711) and monovalent (ASTX660) IAP antagonists were observed to have rapid and potent on-target effects; however, although each of the CRPC lines formed the cell death-inducing Complex-IIb these cell lines were relatively (PC3) or totally (DU145, VCaP) resistant to apoptosis induced by IAP antagonists alone and when co-treated with TNFα or macrophage conditioned media to mimic a proinflammatory TME. TL32711 has been reported to be cIAP1-selective, whereas ASTX660 inhibits both cIAP1 and XIAP^24,39^. However, RNAi-mediated depletion of either cIAP2 or XIAP failed to sensitize the CRPC models to TL32711, and cIAP2 depletion failed to enhance response to ASTX660 (not shown), ruling out IAP redundancy as a mechanism of resistance.

The caspase-8 regulator FLIP has been previously shown by our group to be overexpressed in CRPC^6^. In Complex-IIb, FLIP(L)/procaspase-8 heterodimers are partially processed (to p43-FLIP(L) and p41/p43-caspase-8), and this heterodimer has enzymatic activity but cannot initiate cleavage of executioner procaspases and so does not promote apoptosis induction. When FLIP(S) heterodimerizes with procaspase-8 in Complex-IIb, the heterodimer has no enzymatic activity and procaspase-8 cleavage is completely blocked, inhibiting apoptosis, but also potentially promoting necroptosis as RIPK1 is also not cleaved^40^. We found that while selective RNAi-mediated depletion of either FLIP(L) or FLIP(S) enhanced cell death induced by IAP antagonists, depletion of both splice forms was required for the maximal induction of cell death. Notably, we found that cytoplasmic FLIP(L) was highly stable in CRPC cells, with no depletion up to 24 h after siRNA transfection (reported half-life ~3 h^41^); the reason for this hyper-stability is currently under investigation. However, nuclear FLIP(L) was effectively depleted, and its downregulation along with downregulation of the predominantly cytoplasmic FLIP(S) was sufficient to promote apoptosis in response to IAP antagonists. This suggests that in CRPC at least, the nuclear pool of FLIP(L) is more important than the cytoplasmic pool for blocking Complex-IIb-mediated cell death. This was further supported by the observation that the nuclear export inhibitor Leptomycin-B enhanced cell death induced by TL32711/TNFα.

HDAC inhibitors have a wide range of anti-cancer activities primarily through their abilities to modulate gene expression via acetylation of histones and non-histone proteins^35,42^. Because of these activities, HDAC inhibitors are being pre-clinically and clinically investigated in a number of cancers in combination with other agents, most notably immune oncology agents^43,44^. We and others have previously demonstrated that HDAC inhibitors downregulate FLIP expression by both transcriptional and post-transcriptional mechanisms^33,45,46^. The Class I-selective HDACi Entinostat has been given Breakthrough Therapy status by the FDA after showing promising results in the treatment of aromatase inhibitor-resistant breast cancer^32^. Because of this activity in a hormone-resistant disease setting, we explored Entinostat as a clinically relevant approach for targeting FLIP expression in CRPC to overcome resistance to IAP antagonists. Entinostat was observed to downregulate nuclear FLIP(L) and cytoplasmic FLIP(S) protein expression via post-transcriptional mechanisms in two out of three CRPC models and subsequently sensitize these models to IAP antagonist therapy in vitro and in vivo. Mechanistically, this appears to be due to Entinostat-induced acetylation of Ku70, a protein best characterized as a key component of the DNA damage repair machinery^47^, which we have previously shown to inhibit FLIP ubiquitination and degradation in an acetylation-dependent manner^33^. In prostate cancer cells, Ku70 was found to be acetylated in response to Entinostat, and siRNA-mediated Ku70 depletion resulted in downregulation of nuclear but not cytoplasmic FLIP(L). Gong *et al*. proposed a similar mechanism by which HDAC inhibition induces an acetylation-dependent disruption of the Ku70:FLIP complex^48^. In addition, FLIP(L)/(S) downregulation following Entinostat treatment may also be caused by activation of JNK activity^49^. JNK could subsequently activate the E3-ubiquitin ligase ITCH, which has previously been reported to promote FLIP degradation via the ubiquitination-proteasome system (UPS)^50^.

Entinostat-mediated enhancement of TL32711/TNFα-induced cell death was also determined to be caspase-dependent (i.e., not necroptotic) and, more specifically, caspase-8-dependent. We also demonstrated that the cell death induced by TL32711/TNFα in the presence and absence of Entinostat was dependent on RIPK1 and FLIP’s ability to interact with the key Complex-IIb adaptor protein FADD. Thus, the sensitizing effects of Entinostat are clearly due to its effects on FLIP rather than other effects of Class-I HDAC inhibition. In conclusion, these results show that although IAP antagonists promote formation of Complex-IIb in proinflammatory models of CRPC, these complexes fail to activate cell death because of the co-recruitment of FLIP. However, the inhibitory effects of FLIP can be overcome using the clinically relevant HDAC inhibitor Entinostat, suggesting that strategies combining this agent with IAP antagonists (particularly better tolerated next-generation antagonists such as ASTX660) may be effective in proinflammatory CRPC.

## Electronic supplementary material

Supplementary figure legends and methods

Supplementary figures 1-6
