# Supplementary material for: Cytoplasmic FLIP(S) and nuclear FLIP(L) mediate resistance of castrate-resistant prostate cancer to apoptosis induced by IAP antagonists
Source: Cell Death Dis. 2018 Oct 22;9(11):1081. doi: 10.1038/s41419-018-1125-5 (PMC6197283; doi:10.1038/s41419-018-1125-5)
Supplement: Supplementary file 1 — Supplementary figure legends and methods [file 41419_2018_1125_MOESM1_ESM.docx]

**Supplementary Information**

**Supplementary Figure Legends:**

**Supplementary Figure 1: *PCa cell lines do not secrete autocrine TNFα***

(**A**) Human TNFα ELISA quantifying TNFα secretion from THP1, M1-differentiated THP1 (as a positive control), PC3 and DU145 cells untreated (Con) and following 24 hours treatment with 1μM ASTX660 or TL32711. (**B**) Standard curve used for quantification of samples.

**Supplementary Figure 2: *On target activity of ASTX660***

(**A**) Western blot analysis of RIPK1 and caspase-8 following caspase-8 immunoprecipitation in PC3 and DU145 cell lines treated for 3 hours with 1µM ASTX660 or 1µM TL32711(TL) +/-10 ng/ mL TNFα; cells were pretreated with 20µM z-VAD-fmk. (**B**) Cell viability assay in PC3 and DU145 cells treated for 72 hours with 10nM, 100nM, 1μM or 10μM ASTX660 +/-10 ng/ mL TNFα.

**Supplementary Figure 3: *FLIP siRNA sensitises to TL32711+TNFα***

Cell viability assay of PC3, DU145 and VCaP cells treated for 24h with 10nM scrambled control (SC) siRNA, FLIP(S) specific (FS) siRNA, FLIP(L) specific (FL) siRNA or siRNA targeting both FLIP(L) and FLIP(S) spliceforms (FT) siRNA followed by 48h treatment with 1μM TL32711(TL) +/-10ng/mL TNFα.

**Supplementary Figure 4: *FLIP siRNA sensitises to ASTX660***

**(A**) Annexin-V/Propidium Iodide flow cytometry analysis in PC3 and DU145 cells treated for 24 hours with 10nM scrambled control (SC) or siRNA targeting both FLIP(L) and FLIP(S) spliceforms (FT) siRNA followed by 24 hours treatment with 1µM ASTX660(ASTX) +/-10 ng/ mL TNFα. **(B**) Cell viability assay in PC3 and DU145 cells treated for 24h with 10nM SC or siRNA targeting both FLIP(L) and FLIP(S) spliceforms (FT) siRNA followed by 48h with 1μM ASTX660(ASTX) alone or in combination with 10ng/mL TNFα.

**Supplementary Figure 5: *Entinostat post-translationally downregulates FLIP***

Quantitative RT-PCR analysis of FLIP(L) *(Top)* and FLIP(S) *(Bottom)* mRNA expression in PC3 and DU145 cells following 24 hours treatment with 1μM Entinostat. Values presented as delta-delta (ΔΔ)CT ratio normalized to RPL24 housekeeping gene.

**Supplementary Figure 4: *Entinostat sensitises to ASTX660***

(**A**) Annexin-V/PI flow cytometry analysis of PC3, DU145 cells upon 24 hours pre-treatment with 1μM Entinostat (Entin) followed by 24 hours with 1µM ASTX660(ASTX) +/-10ng/mL TNFα. (**B**) Cell viability assay in PC3 and DU145 cells following 24 hours pre-treatment with 0, 0.1, 0.5, 1.0 and 2.5μM Entinostat followed by 48 hours with 1µM ASTX660(ASTX) +/-10ng/mL TNFα.

**Supplementary Methods:**

**Supplementary Methods 1: *Subcellular Fractionations***

Nuclear and cytoplasmic fractions were isolated by lysing the cells for 20 minutes at 4^o^C in ‘Buffer A’ (10mM HEPES pH7.4, 1.5mM MgCl_2_, 10mM NaCl, 0.1% NP40, 1mM PMSF, 0.1mM TLCK, 0.1mM TPCK, 1mM NaF, 1mM Na_3_VO_4_) followed by centrifugation at 4000 RPM for 2 minutes, supernatant was removed and centrifuged for a further 2 minutes; this yields the cytoplasmic fraction. The remaining pellet was subjected to further lysis in ‘Buffer C’ (10mM HEPES pH7.4, 1.5mM MgCl_2_, 420mM NaCl, 0.1% NP40, 1mM PMSF, 0.1mM TLCK, 0.1mM TPCK, 1mM NaF, 1mM Na_3_VO_4_) for 10 minutes at 4^o^C, followed by sonication at 20,000 cycles/sec for 30 seconds. A final centrifugation at 4000 RPM for 2 minutes yielded the nuclear fraction.

**Supplementary Methods 2: *siRNA Sequences***

siRNA sequences are as follows: SC- UUCUCCGAACGUGUCACGU, cIAP2- CAAGAUACACAGUUUCUAATT, XIAP- GAAGGGACAAGAAUAUAUAUU, FLIP(L)- AACAGGAACTGCCTCTACTT, FLIP(S)- AAGGAACAGCTTGGCGCTCAA, FLIP(T)- AAGCAGTCTGTTCAAGGAGCA, RIPK1- GGAGCAAACUGAAUAAUGAUU, Ku70- GAGGATCATGCTGTTCACCAA

**Supplementary Methods 3: *Quantitative PCR***

RNA was extracted using a Roche High Pure RNA Isolation Kit (Roche, Basel, Switzerland), according to manufacturer’s instructions. cDNA was prepared using a Roche Transcriptor First Strand cDNA synthesis kit (Roche, Basel, Switzerland), according to manufacturer’s instructions. Quantitative Real-Time PCR was carried out using Sybr green dye (Roche) and the following primer sequences: FLIP(L): **F**: CCTAGGAATCTGCGTGATAATCGA **R**:TGGGATATACCATGCATACTGAGATG, FLIP(S): **F**:ATTTCCAAGAATTTTCAGATCAGGA **R**:GCAGCAATCCAAAAGAGTCTCA. RPL24 (housekeeping gene): **F:** AAGGCTCAACGAGAACAAGC, **R:** GGTGCTGCCTTTGTAGGTG.
