# Supplementary material for: Cytoplasmic FLIP(S) and nuclear FLIP(L) mediate resistance of castrate-resistant prostate cancer to apoptosis induced by IAP antagonists
Source: Cell Death Dis. 2018 Oct 22;9(11):1081. doi: 10.1038/s41419-018-1125-5 (PMC6197283; doi:10.1038/s41419-018-1125-5)
Supplement: Supplementary file 2 — Supplementary figures 1-6 [file 41419_2018_1125_MOESM2_ESM.pptx]

## Slide 1
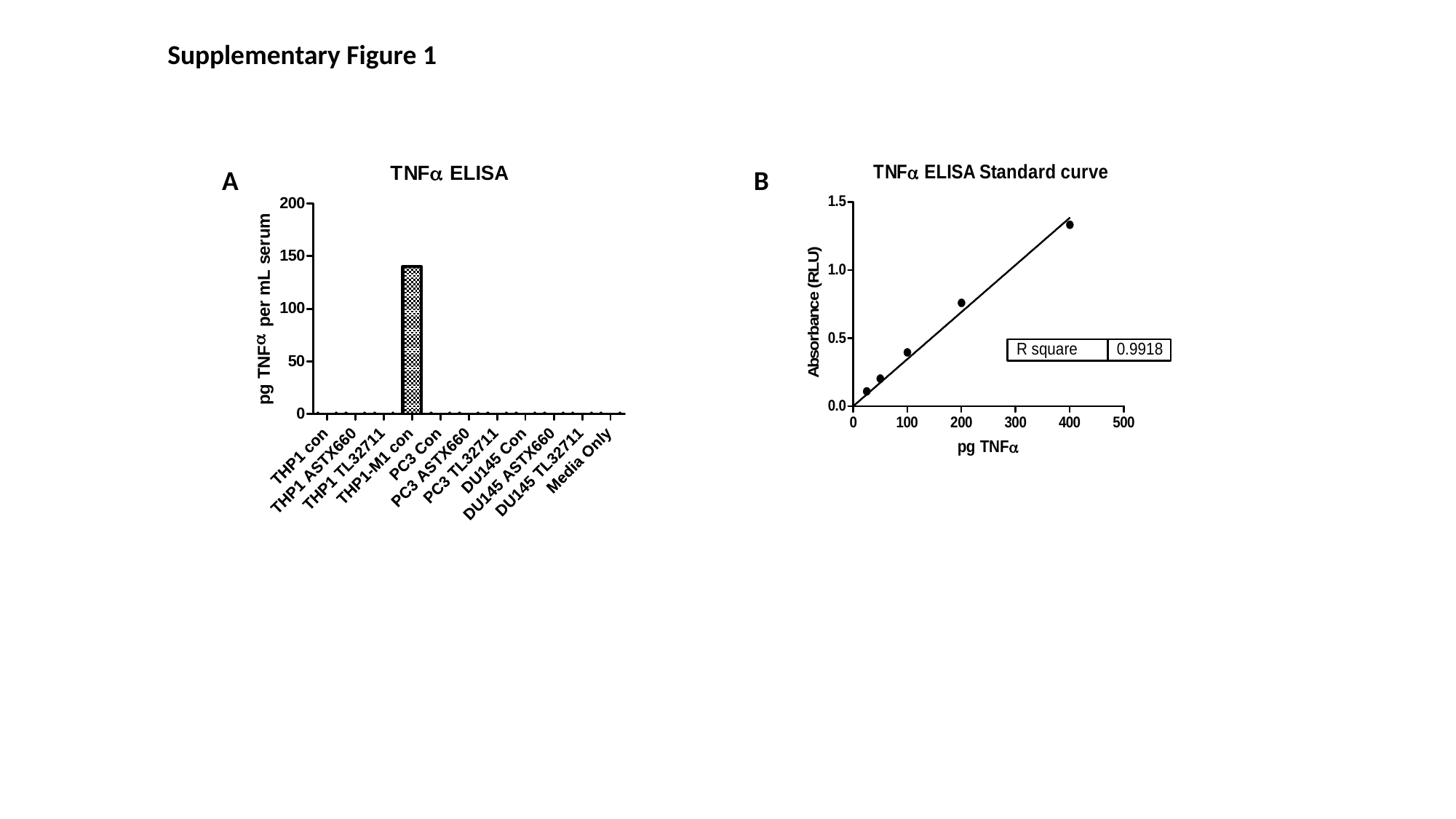

Supplementary Figure 1
A
B

## Slide 2
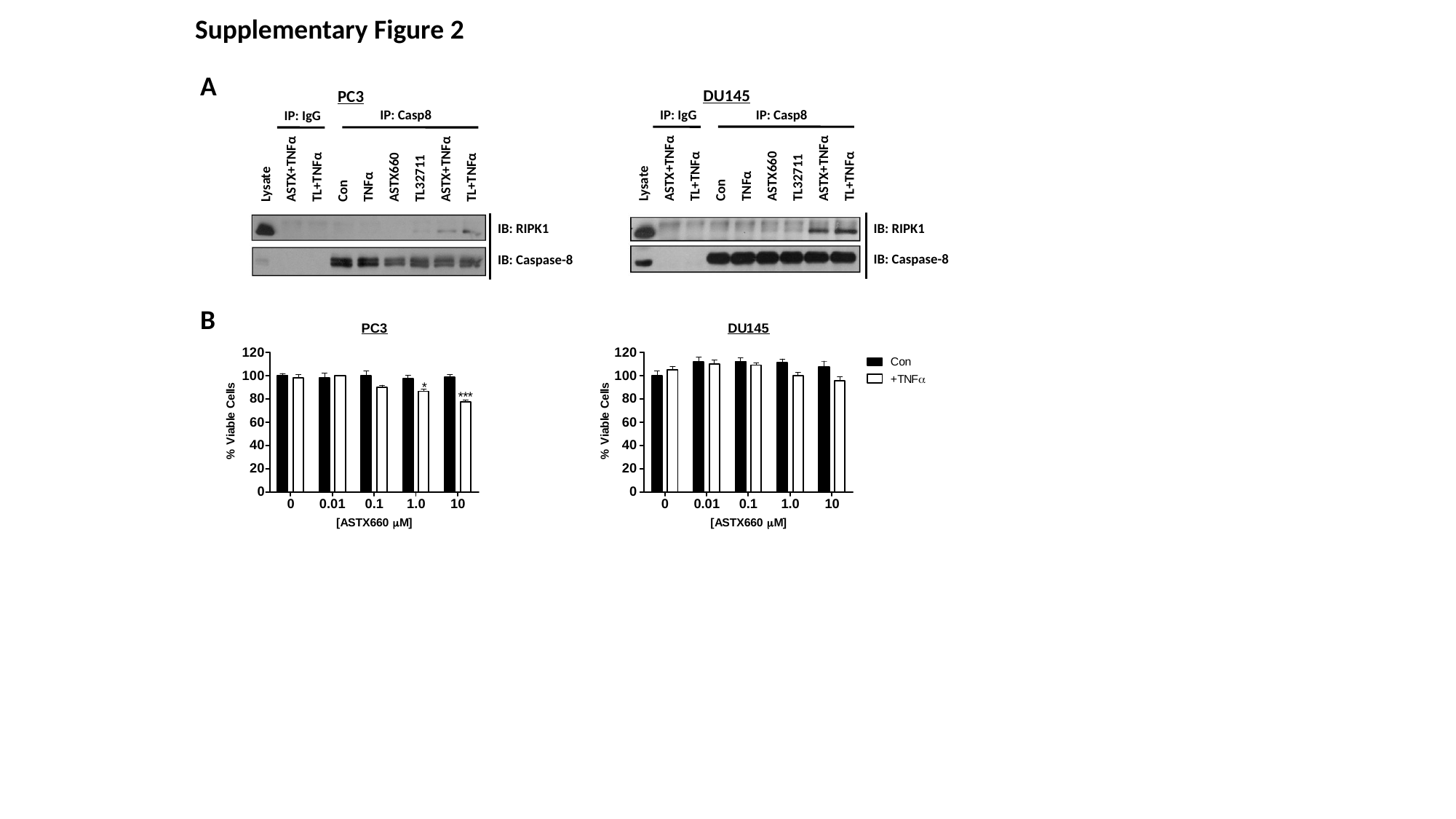

Supplementary Figure 2
A
DU145
ASTX+TNFα
TL+TNFα
Con
TNFα
ASTX+TNFα
TL+TNFα
ASTX660
TL32711
Lysate
IP: Casp8
IP: IgG
IB: RIPK1
IB: Caspase-8
PC3
ASTX+TNFα
TL+TNFα
Con
TNFα
ASTX+TNFα
TL+TNFα
ASTX660
TL32711
Lysate
IP: Casp8
IP: IgG
IB: RIPK1
IB: Caspase-8
B

## Slide 3
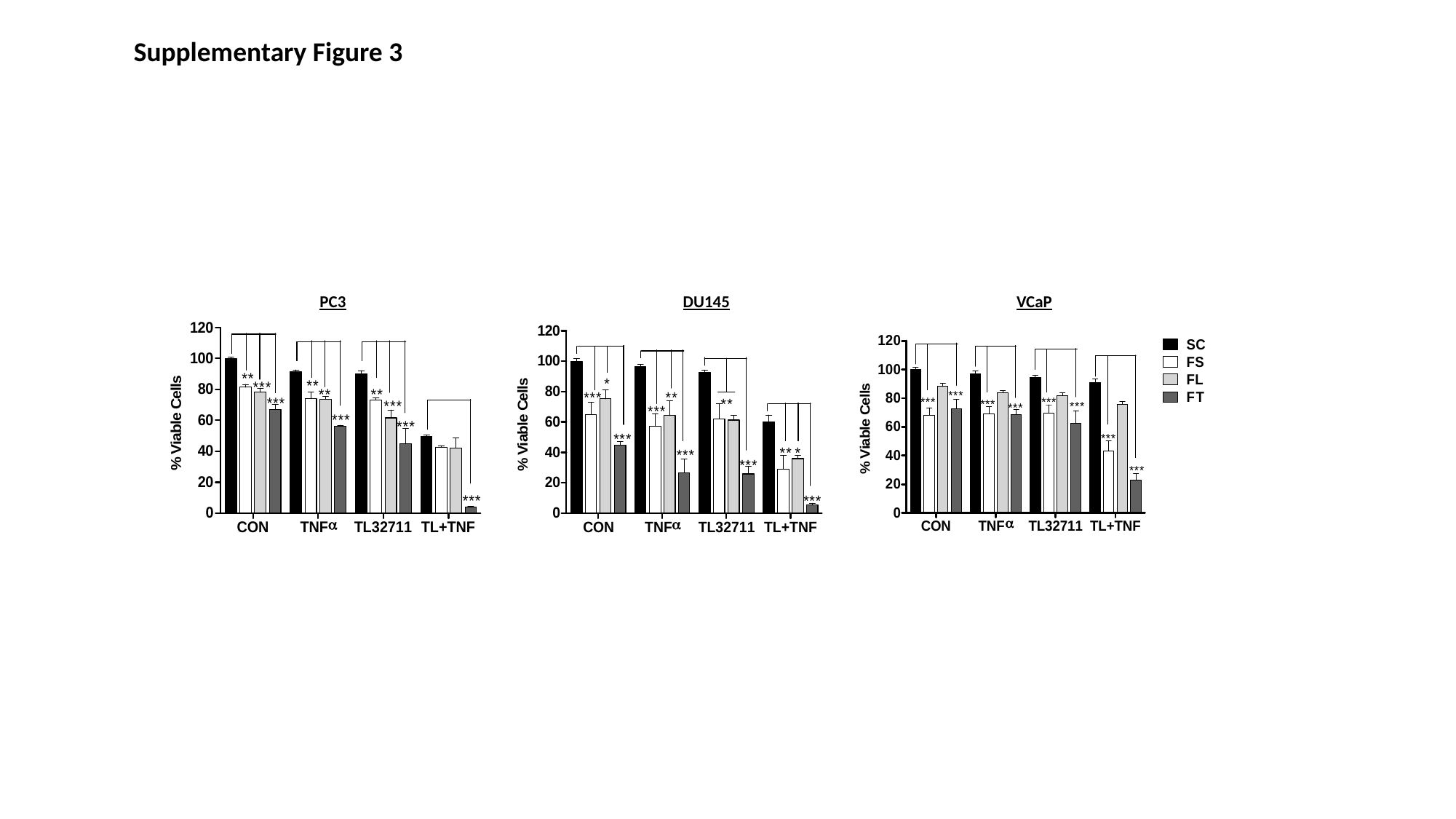

Supplementary Figure 3
PC3
DU145
VCaP

## Slide 4
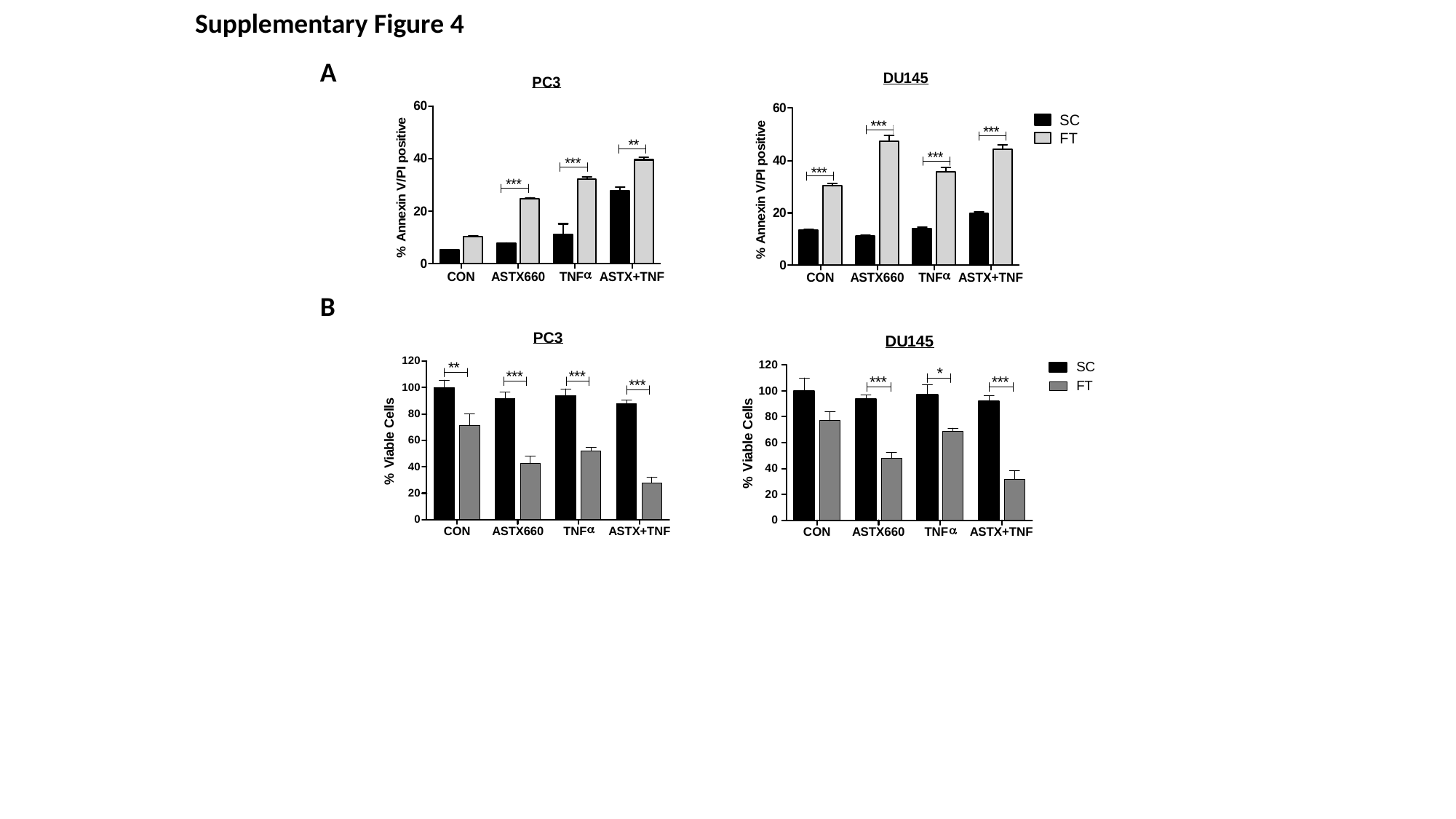

Supplementary Figure 4
A
B

## Slide 5
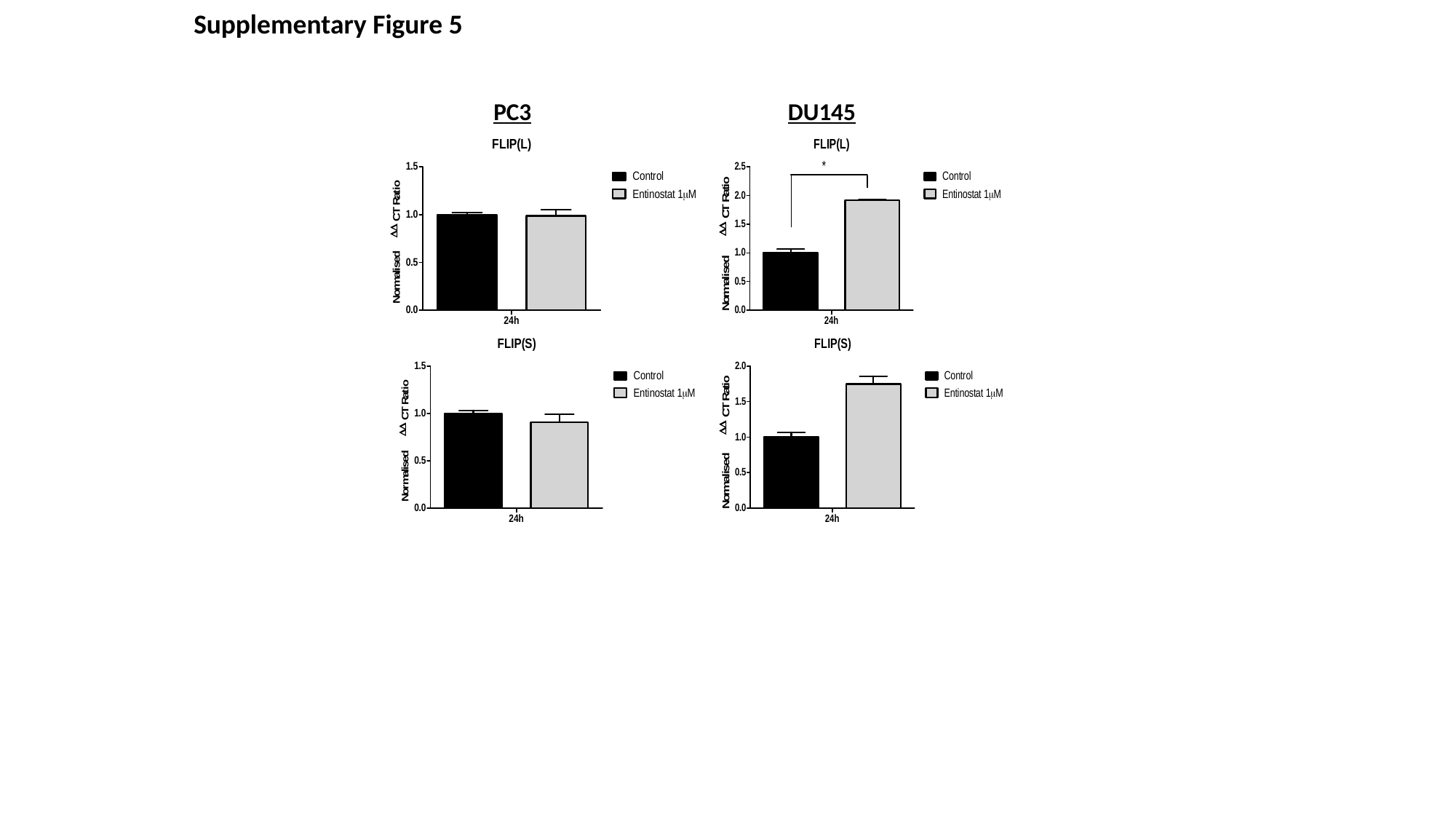

Supplementary Figure 5
PC3
DU145

## Slide 6
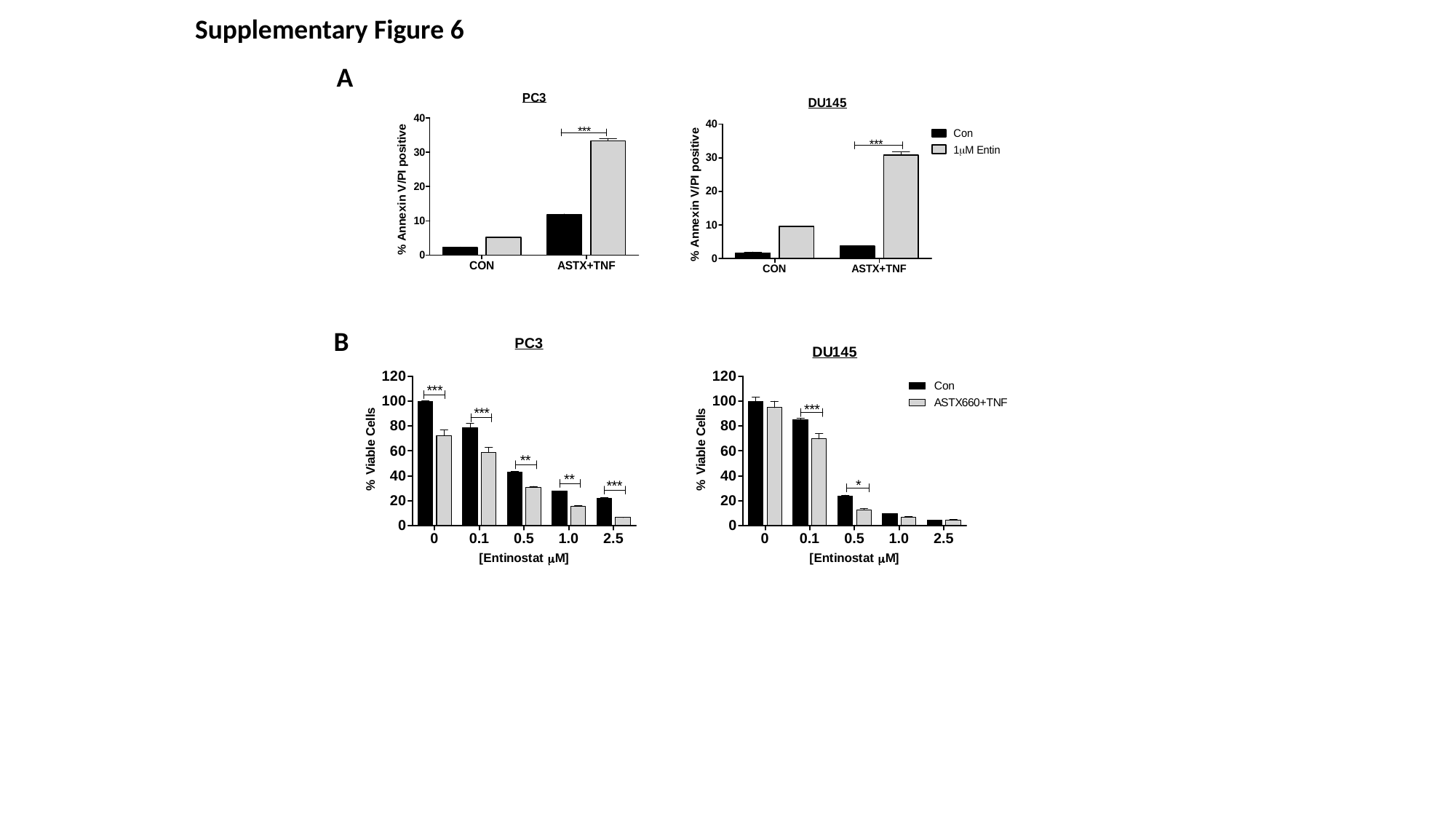

Supplementary Figure 6
A
B
